# Molecular Epidemiology of HIV-1 in Panama: Origin of Non-B Subtypes in Samples Collected from 2007 to 2013

**DOI:** 10.1371/journal.pone.0085153

**Published:** 2014-01-13

**Authors:** Yaxelis Mendoza, Gonzalo Bello, Juan Castillo Mewa, Alexander A. Martínez, Claudia González, Claudia García-Morales, Santiago Avila-Ríos, Gustavo Reyes-Terán, Juan M. Pascale

**Affiliations:** 1 Department of Genomics and Proteomics, Gorgas Memorial Institute for Health Studies, Panama City, Panama; 2 Department of Biotechnology, Acharya Nagarjuna University, Guntur City, Andhra Pradesh, India; 3 Department of Genetics and Molecular Biology, University of Panama, Panama City, Panama; 4 INDICASAT-AIP, 219, City of Knowledge, Clayton, Panama City, Panama; 5 Laboratório de AIDS e Imunologia Molecular, Instituto Oswaldo Cruz, FIOCRUZ, Rio de Janeiro, Brazil; 6 Centro de Investigación en Enfermedades Infecciosas, Instituto Nacional de Enfermedades Respiratorias, Mexico City, Mexico; Institut Pasteur of Shanghai, Chinese Academy of Sciences, China

## Abstract

Phylogenetic studies have suggested that the HIV-1 epidemic in the Americas is mainly dominated by HIV subtype B. However, countries of South America and the Caribbean have recently reported changes in their circulating HIV-1 genetic profiles. The aim of this study was to characterize the molecular profile of the HIV-1 epidemic in Panama by the analysis of 655 polymerase gene (*pol*) sequences that were obtained from HIV-infected Panamanians diagnosed between 1987 and 2013. Blood samples were collected from recently infected, antiretroviral drug-naïve and treatment-experienced subjects since mid-2007 to 2013. Viral RNA from plasma was extracted and sequences of HIV protease and reverse transcriptase genes were obtained. Bootscanning and phylogenetic methods were used for HIV subtyping and to trace the putative origin of non-B subtype strains. Our results showed that HIV-1 infections in Panama are dominated by subtype B (98.9%). The remaining 1.1% is represented by a diverse collection of recombinant variants including: three URFs_BC, one CRF20_BG, and one CRF28/29_BF, in addition to one subtype F1 and one subtype C, none of which were previously reported in Panama. The non-B subtype variants detected in Panama were probably introduced from Brazil (subtype F1 and CRF28/29_BF), Cuba (CRF20_BG), Dominican Republic (URFs_BC) and India (subtype C). Panama is the geographical vertex that connects the North with South America and the Caribbean through trade and cultural relations, which may explain the observed introductions of non-B subtype HIV-1 variants from both the Caribbean and South America into this Central American country.

## Introduction

The Human Immunodeficiency Virus (HIV) is the causative agent of Acquired Immune Deficiency Syndrome (AIDS). During the past decade, the Panamanian HIV/AIDS epidemic has changed dramatically. The accumulated number of adults (15+ years) living with HIV increased from 5,000 to around 18,000–20,000 subjects between the years 2001–2010 [1], [2], whereas the number of newly infected people increased up to 14.9% (from 440 to 942 cases) in the same period [3], [4]. At the beginning of the Panamanian epidemic, the majority of the infections had occurred between men who have sex with men (MSM); although, since 1991, there has been an increase in the number of infected women [5], [6]. Nowadays, recent studies have suggested that the Panamanian epidemic does not have a predominant mode of sexual transmission because bisexual-homosexual and heterosexual modes have similar proportions [2]. Finally, even though the total HIV prevalence has decreased from 1.4% to 0.8% in the general population since 2001 and the prevalence among female sex workers remains low (0.7–1.6%) [7], the prevalence among MSM continues to steadily increase and reached 23% in 2011 [8]. A higher prevalence of HIV infections among MSM in the Americas was only observed in Jamaica (38%) [8].

The main characteristic of HIV infection is the high genetic variability of virus isolates obtained either sequentially from the same infected subject or from different subjects [9]–[11]. Phylogenetic studies allow the classification of HIV-1 isolates into four main groups (M, N, O and P) [12]. Currently, HIV-1 group M is responsible for most HIV-1 infections worldwide and has been classified into nine subtypes (A–D, F–H, and J–K), 55 circulating recombinant forms (CRFs) and a large number of unique recombinant forms (URFs) [13], [14]. HIV-1 subtype B is the predominant variant in the Americas; although non-B subtypes have also been described with high prevalence in Cuba, Brazil, Argentina, and Uruguay [15]. Panamás geographical position and its historical mission as a country of transit since the colonial era have made the country a highly cosmopolitan nation with close historical and cultural links with Central, North and South America and the Caribbean. Therefore, the genetic diversity of HIV-1 in Panama is expected to resemble the diverse genetic profile observed in other parts of the Americas and the Caribbean. The only other HIV-1 molecular epidemiologic survey performed in Panama to date, analyzed the *gag*, *pol*, and *env* genes of 133 samples collected between 2004 and 2005, reporting a high prevalence of subtype B (97%) and only two cases of non-B-subtypes (one CRF12_BF and one CRF02_AG) [16], [17]. The aim of our study was to expand our comprehension of the HIV-1 subtype distribution in Panama by the phylogenetic analysis of the *pol* region of a large number of individuals (*n*  =  655) from different regions of the country that were diagnosed over a long time period (1987–2013).

## Materials and Methods

### Study population

Blood samples from HIV-1 seropositive individuals were collected by venipuncture at Gorgas Memorial Institute or received from local hospitals located at different provinces of Panama between mid-2007 to June 2013. HIV-1 *pol* region sequences were obtained using the Viroseq system (Celera Diagnostics, Alameda, CA) or an “in-house” assay (described below) drug resistance genotyping method. Sequence chromatograms from a total of 754 samples were retrieved from the HIV-1 Resistance Genotyping Test computers and carefully re-evaluated with Sequencher Software, version 4.5 (GeneCodes, Michigan, USA). From the 754 sequences obtained, 655 (404 sequences from Viroseq system and 251 sequences from the “in-house” assay) were selected based on sequence length and only one sequence per studied subject. The epidemiological information from the selected subjects was recovered from the Drug-Resistance Genotyping Test form, sent with the sample by an authorized infectious diseases specialist.

### Ethics approval and patient consent

The study cohort included subjects selected from the National Surveillance System (*n*  =  466) and recruited from a research project entitled “Molecular Epidemiology of HIV in the Meso-American Region” (*n*  =  189). The Gorgas Memorial Institutional Review Board approved the use of samples without an informed written consent from subjects coming only from the national epidemiological surveillance system and only for epidemiological purposes. Informed written consent was obtained from subjects who participated in the research project.

### Pol gene sequencing: “in-house” drug resistance genotyping assay

Plasma samples were centrifuged at 20,000 g for 1 hour and viral RNA was extracted using the QIAamp Viral RNA Mini kit (Qiagen Inc., Valencia, CA). Reverse transcription was performed using the Thermoscript Reverse Transcriptase enzyme (Invitrogen, Carlsbad, CA), following the manufacturer’s instructions. A 1.2 kb fragment of the HIV-1 *pol* gene spanning the complete protease (Pro, codons 1–99) and part of reverse transcriptase (RT, codons 1–235) was amplified by a nested polymerase chain reaction (PCR) using Platinum Taq polymerase (Invitrogen, Carlsbad, CA). Both PCR reactions were performed in a final volume of 50 µL with 1.8 mM MgCl2, 0.2 mM dNTP mix, 0.2 µM each primer. The first round PCR was carried out under the following conditions: 94°C, 2min, 30 cycles at 94°C-20s, 50°C-20s, 72°C-90s, final extension of 72°C-6min. Second round PCR conditions were: 94°C, 2min, 40 cycles at 94°C-20s, 50°C-20s, 72°C-90s, final extension of 72°C-6min. PCR products were electrophoresed on 1% agarose gels and DNA bands of expected size purified using AgarAce enzyme (Promega, Madison, WI). Direct cycle sequencing was performed with seven overlapping segment primers using the ABI Prism BigDye Terminator v3.1 Cycle Sequencing kit and an ABI PRISM 3130xl Genetic Analyzer (Life Technologies, Carlsbad, CA). Primers used for PCR and sequencing have been previously described [18]. Sequence fragments were assembled using the Sequencher software version 4.5 (GeneCodes, Ann Arbor, MI).

### Genetic characterization of HIV-1 sequences

Panamanian sequences were initially classified as “pure” subtypes, CRFs-like or URFs using the online web server for REGA HIV-1 Subtyping Tool software (version 2.0) [19]. Initial classification was confirmed using phylogenetic and recombination analysis. Group M subtype (Table S1) and CRFs reference sequences of the *pol* gene (positions 2252 to 3260 relative to HXB2) were downloaded from Los Alamos HIV Sequence Database (http://hiv-web.lanl.gov) and aligned with Panamanian sequences using the ClustalW program implemented in Mega 5.1 software [20]. Phylogenetic trees were constructed by the Neighbor-Joining method under the Tamura-Nei evolutionary model using the MEGA 5.1 software package [20]. The reliability of tree topologies was assessed by bootstrap analysis with 500 replicates. Bootstrap values above 75% were considered significant. Analysis of recombination was initially performed by bootscan analysis as implemented in the Simplot version 3.5.1 [21] using representative of all HIV-1 group M subtypes as reference. Bootstrap values supporting branching with reference sequences were determined in Neighbor-Joining trees constructed using the K2-parameter model, based on 500 replicates, with a 250bp sliding window moving in steps of 10 bases. To better characterize the recombination breakpoints suggested in the previous analyses, the putative recombinants were subjected to informative site analyses as described elsewhere [22]. Fragments of sequences assigned to specific HIV-1 subtypes were finally confirmed by constructing phylogenetic sub-region trees as previously indicated using MEGA 5.1 software.

### Determination of the origin of non-B subtype HIV-1 variants

To determine the most probable geographic origin of the minor HIV-1 genetic variants circulating in Panama, each non-subtype B Panamanian sequence was aligned with the 50 HIV-1 sequences isolated world-wide with the highest BLAST search similarity score and subject to Maximum Likelihood (ML) phylogenetic analysis using the GTR+I+Γ nucleotide substitution model. The ML tree was reconstructed with the PhyML program [23] using an online web server (http://www.atgc-montpellier.fr/phyml/). Heuristic tree search was performed using the SPR branch-swapping algorithm and the reliability of the obtained topology was estimated with the approximate likelihood-ratio test (aLRT) [24] based on the Shimodaira-Hasegawa-like procedure. The ML trees were visualized using the FigTree v1.4.0 program (http://tree.bio.ed.ac.uk/software/figtree/).

### Statistical analysis

Epidemiological and demographic characteristics of the cohort included in the present study were compared with overall characteristic of the officially reported HIV cases by the Department of Epidemiology of the Ministry of Health (MINSA) [1], [25] using a Two-sample proportion test. Statistical significance was defined as *p*<0.05.

### Nucleotide Sequence Accession Numbers

The non-subtype B sequences have been deposited in Genbank with accession numbers KF702320 - KF702326.

## Results

### Epidemiological characteristics

**Figure 1:**
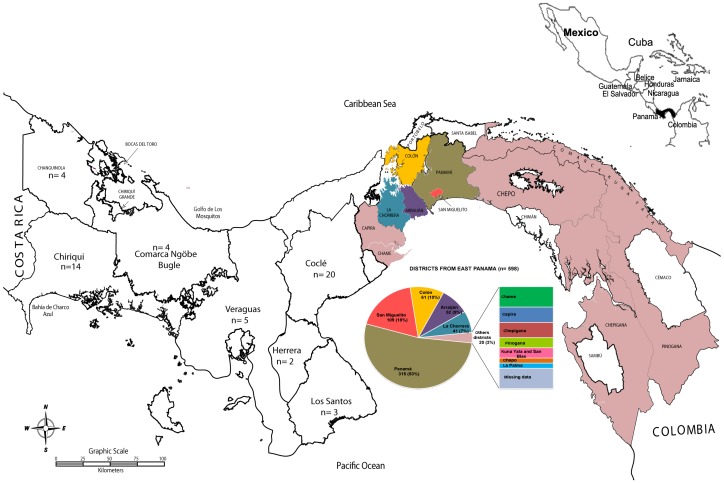
Geographic distribution of Panamanian subjects living with HIV participating in the study. Map of Panama indicating the number of infected subjects located in each of the nine provinces (Bocas del Toro, Chiriquí, Veraguas, Herrera, Los Santos, Coclé, Colón, Panamá and Darién) and native territories of Comarcas Ngöbe Buglé, Kuna Yala and Emberá Wouman. The pie chart that represents the proportion of subjects located at the different districts from eastern Panama which includes the provinces of Panama, Colón, Darién and Comarca Kuna Yala.

The 655 HIV sequences analyzed in our study represent 3.6% of the total estimated number of HIV-infected subjects and 6% of the total number of ARV drug-experienced subjects in Panama who have accessed the National Health System during 2007–2013. In our study, most HIV-infected individuals in the adult population (15+ years) were men (62%), asymptomatic (54%), and had a diagnosis during 2005–2009 (35%) (Table 1). The male to female ratio was 2:1 (408/194) in the adult population and 1.2:1 (28/25) in the pediatric population. The main mode of transmission was sexual intercourse (324/655, 49%), followed by mother-to-child transmission (55/655, 8%). Subjects were located in the nine provinces of Panama and the native autonomous territories of Comarca Kuna Yala and Comarca Ngöbe Bugle, although the majority of the subjects were living in the Province of Panama (527/655; 80%) and Colon (61/655, 10%) (Fig. 1). Statistical analyses show that subjects located in the eastern part of Panama and pediatric subjects acquiring the infection by mother-to-child transmission were found to be significantly different from national reported data (Table 1). However, the others epidemiological characteristics (sexual transmission, clinical condition, gender proportions, age groups and geographic locations), were not statistically different to those found in the national records of HIV-infected subjects (Table 1). Therefore, the cohort included in the present study have these others epidemiological characteristics similar to total population of HIV-infected subjects.

**Table 1:** Demographic and clinical characteristics of the study population compared to National Governmental Report of Panamanian HIV cases (1984–2012).

		Present Study (n = 655)	Overall Panamanian HIV reported cases (n = 18.700)a	p value*
**Gender**				
	Male	436 (66.6)	12,973 (69.4)	n.s.
	Female	219 (33.4)	5,730 (30.6)	n.s.
**Clinical Condition**				
	AIDS	185 (28.2)	-	
	Asymptomatic HIV carrier	355 (54.2)	-	
	Unknown	115 (17.56)	-	
**Mode of HIV Transmission**				
Sexual		324 (49.5)	11,361 (60.7)	p<0.05
	Homosexual	65 (9.2)	1,940 (10.4)	n.s.
	Bisexual	4 (0.6)	690 (3.7)	n.s.
	Heterosexual	255 (38.9)	8,731 (40.7)	n.s.
Blood Products		6 (0.9)	178 (1.0)	n.s.
Mother-to-child		55 (8.3)	564 (3.0)	p<0.05
Unknown		270 (41.2)	6,681 (35.7)	n.s.
**Age group (years)**				
	< 14	53 (8.1)	633 (3.4)	n.s.
	15–24	119 (18.2)	2,530 (13.5)	n.s.
	25–29	66 (10.1)	2,697 (14.4)	n.s.
	30–34	97 (14.8)	2,993 (16.0)	n.s.
	35–39	101 (15.4)	2,688 (14.4)	n.s.
	40–44	75 (11.4)	2,318 (12.4)	n.s.
	45–49	63 (9.6)	1,654 (8.8)	n.s.
	>50	81 (12.4)	3,666 (19.6)	n.s.
	Unknown	0	430 (2.3)	
**Geographic Location**				
	East Panama	598 (91.3)	14,898 (83.5)b	p<0.05
	Central Panama	30 (4.5)	920 (5.2) b	n.s.
	West Panama	22 (3.4)	985 (5.5) b	n.s.
	Unknown	5 (0.8)	1,043 (5.8) b	
**HIV diagnosis**				
	1987–1994	11 (1.7)	-	
	1995–1999	36 (5.5)	-	
	2000–2004	148 (22.6)	-	
	2005–2009	259 (39.5)	-	
	2010–2013	191 (29)	-	

Data are No. (%). Abbreviations: HIV, human immunodeficiency virus; n.s. not significant differences; p<0.05, means significant differences.

p value of the two-sample proportion Z-test result.

^a^ As indicated by the total of accumulative AIDS and HIV carriers first time diagnosed between September 1984 to September 2012.

^b^ As indicated by the total of accumulative AIDS and HIV carriers first time diagnosed between 1984 to 2010.

### Genetic characterization of HIV-1 sequences

**Figure 2:**
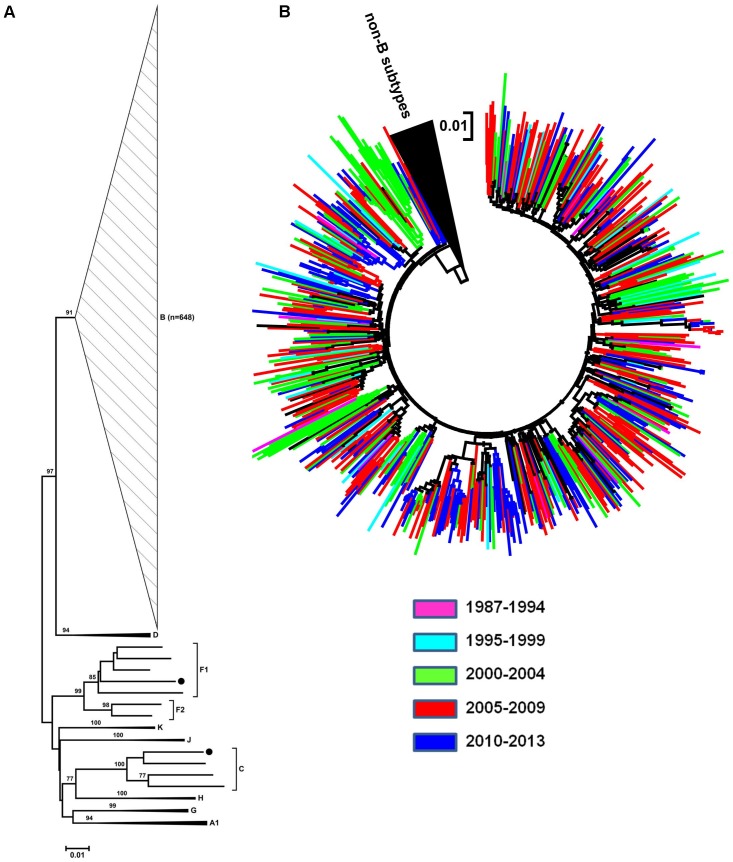
Phylogenetic Neighbor-Joining tree of HIV *pol* gene sequences from Panamanian infected subjects sampled from 2007 to 2013. (A) Panamanian HIV (black circles) are clustered with highly support within the clade of subtype B (n = 648), subtype F1 (n = 1) and subtype C (n = 1) reference sequences. For clarification purposes, branches have been compressed by each subtype. Bootstrap values higher than 80 are shown at branches. (B) Clustering of subtype B sequences defined by colored range of diagnostic age.

**Figure 3:**
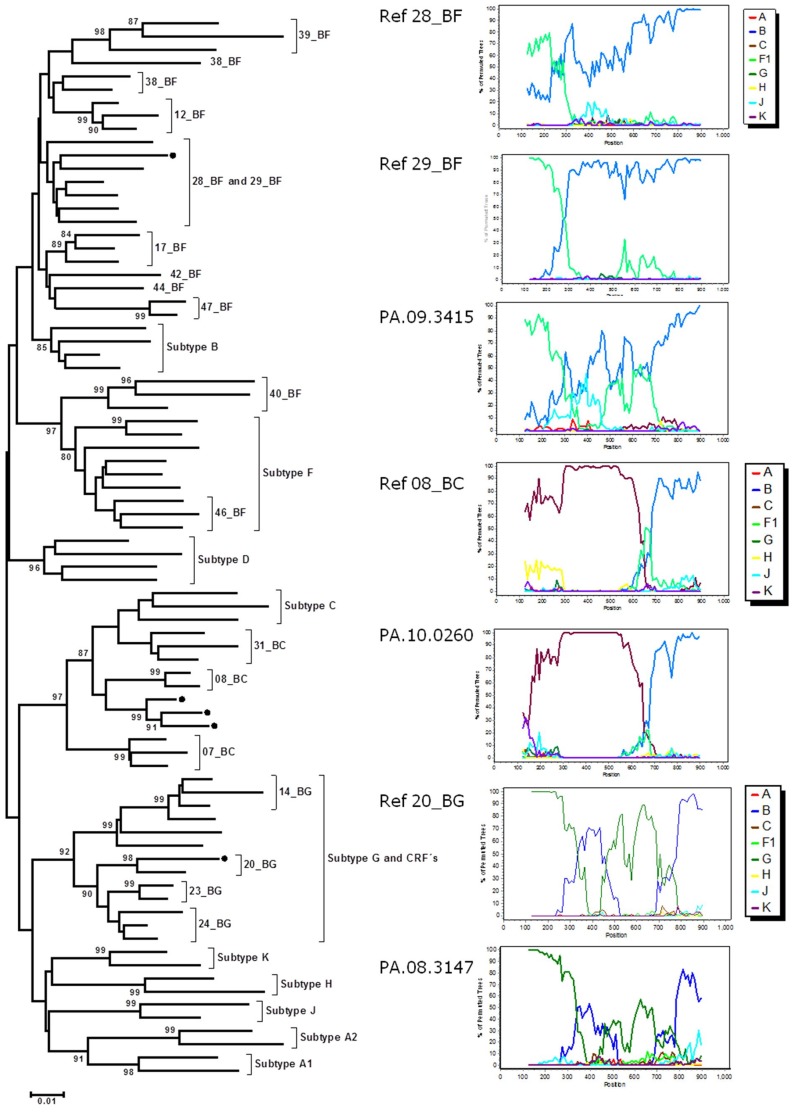
Phylogenetic Neighbor-Joining tree of HIV *pol* gene sequences from Panamanian HIV recombinant variants BF1, BC and BG clustered with circulating recombinant forms (CRF) references. Subtypes and CRF references are indicated at right of the brackets. Only bootstrap values of more than 80 are shown at the corresponding nodes. Panamanian isolates are shown with black circles. For clarity purposes, branches have been compressed by each subtype. Bootscanning plots of these recombinants are shown aside. Reference sequences used for bootscanning analyses shown by color (subtype B, blue; subtype F1, green; subtype C, purple; subtype G, dark-green and subtype A1, red).

Phylogenetic analyses showed that the majority of the HIV-1 sequences were subtype B (648/655, 98.9%) (Fig. 2A). Panamanian subtype B sequences from individuals diagnosed at different time-points were highly intermixed in the phylogenetic tree, with no evidence of expansion of particular lineages among the most recently diagnosed subjects (Fig. 2B). The remaining samples were classified as subtype F1 (0.15%), subtype C (0.15%), BC (0.5%), BF (0.15%) and BG (0.15%) (Figs. 2A and 3). From the seven non-B variants, all the recombinants (BC, BG and BF) were generated using the “in-house” resistance genotyping assay; whereas the subtypes C and F sequences were generated using Viroseq System. Phylogenetic and bootscan analysis revealed that Panamanian recombinant strains displayed similar recombinant structure and branched together with CRFs 08_BC, 20_BG and 28/29_BF references sequences (Fig. 3). Informative site analysis of the breakpoints positions revealed that HIV-1 Panamanian BF and BG recombinants displayed the same mosaic structure as the CRFs 28/29_BF and 20_BG reference sequences, respectively (Table 2). Panamanian BF and BG recombinants strains also branched together with the corresponding CRFs reference strains in all sub-region phylogenetic trees (Figs. S1 and S2), thus confirming their classification as CRF 20_BG-like and CRF 28/29_BF-like strains. The three Panamanian BC recombinants share the first breakpoint position in common with reference CRF08_BC, but not the second one (Table 2). Phylogenetic analysis of each sub-region showed that Panamanian BC and CRF08_BC strains branched separately in the subtype B fragment (Fig. S3), thus confirming their classification as URFs_BC. Epidemiological data on each of the non-B subtypes is shown in Table 3.

**Table 2:** Comparative analysis of the breakpoints positions between HIV-1 Panamanian recombinants sequences and the most closely related CRFs sequences.

	Los Alamos		Our study	
Sequence Name/accession number	Position	Subtype	Position	Subtype
CRF 08_BC AY008715/HM067748	2253–2852	C	2253–2854	C
	2853–3149	B	2855–3167	B
	3150–3275	C	3168–3275	C
BC_PA.10.0260	-	-	2253–2854	C
	-	-	2855–3275	B
BC_PA.10.5652	-	-	2253–2854	C
	-	-	2855–3275	B
BC_PA.12.0103	-	-	2253–2854	C
	-	-	2855–3275	B
CRF 28_BF DQ085872/DQ085873/DQ085874	2253–2571	F1	2253–2574	F1
	2572–3275	B	2575–3275	B
CRF 29_BF DQ085871/DQ085876/AY771590	2253–2571	F1	2253–2574	F1
	2572–3275	B	2575–3275	B
BF_PA.09.3415	-	-	2253–2574	F1
			2575–3275	B
CRF 20_BG AY586545/AY586544/DQ020274	2253–2551	G	2253–2595	G
	2552–2740	B	2596–2736	B
	2741–2974	G	2737–2994	G
	2975–3181	B	2995–3171	B
	3182–3275	G	3172–3275	G
BG_PA.08.3147	-	-	2253–2595	G
			2596–2736	B
			2737–2994	G
			2995–3171	B
			3172–3275	G

**Table 3:** Epidemiological information of Panamanian subjects with non-B subtypes and recombinant variants BG, BC and BF.

	BG	BF	BC	BC	BC	Subtype F1	Subtype C
Sample name	PA-3147	PA-3415	PA-0260	PA-5652	PA-0103	PA-P098	PA-4704
**Gender**	M	M	M	F	F	M	F
**Age**	25	30	48	36	37	47	56
**Diagnosis date**	2008	2009	2009	2007	1998	2007	2006
**Mode of transmission**	U	U	U	HE	HE	HE	U
**Clinical condition**	ASY	U	AIDS	ASY	AIDS	AIDS	U
**Geographic area**							
**(Province**	Chiriquí/	Panamá/	Colón/	Panamá/	Panamá/	Panamá/	Panamá/
**/District)**	Boquete	Panamá	Colón	La Chorrera	Panamá	Panamá	San Miguelito
**Patient drug status**	NA	U	NA	EX	EX	EX	EX
**Time under ARV treatment (years)**	-	U	-	2	11	U	7
**Viral Load (copies/ml)**	518	3220	4311023	36358	5230	121564	117615
**Presence of mutations to ARV drug inhibitors**	none	Minor protease	none	none	NRTI and NNRTI	Minor protease	NRTI and NNRTI

Abbreviations: M, male; F, female; U, not known; HE, heterosexual; ASY, asymptomatic; AIDS, acquired inmunedeficiency syndrome; NA, naïve; EX, experienced; NRTI, nucleosidic reverse transcriptase inhibitor; NNRTI, non-nucleosidic reverse transcriptase inhibitor.

### Origin of non-B subtype HIV-1 variants

**Figure 4:**
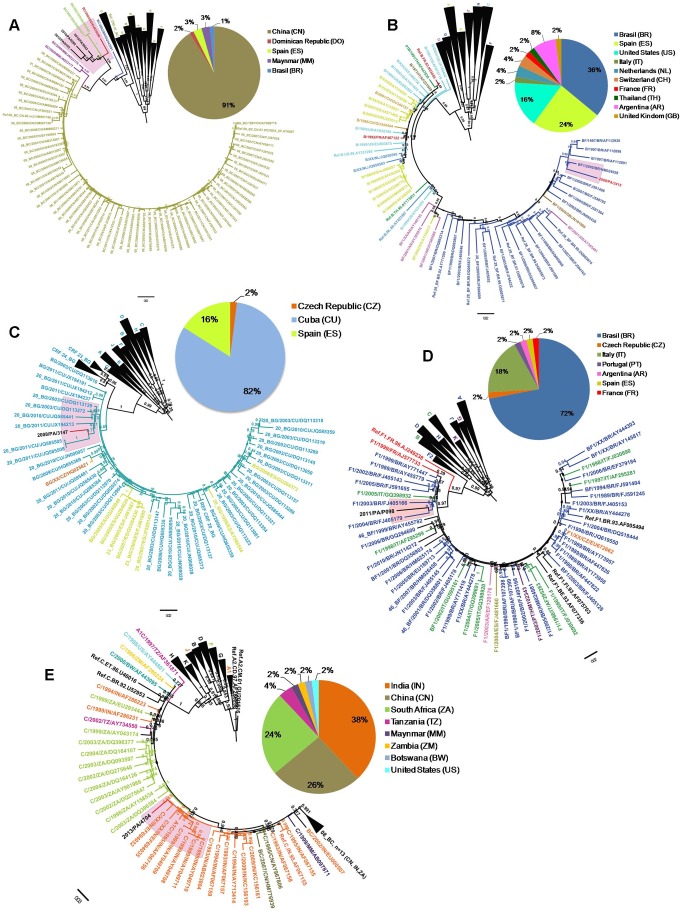
*pol* gene ML phylogenetic tree of non-B HIV-1 Panamanian sequences and 50 highly similar (nucleotide similarity > 94%) sequences from other countries selected with Blastn analyses. (A) BC recombinant. (B) BF1 recombinant. (C) BG recombinant. (D) subtype F1 (E) subtype C. Panamanian non-B sequences are shown in highlighted red color. The tree was rooted using reference subtype sequences. The numbers along branches correspond to aLRT values. Bar is in nucleotide substitutions per site. The pie chart shows the proportion of countries represented by the Blastn analysis. Countries correspond with defined colors which are specified in each pie chart.

ML phylogenetic analyses of non-subtype B Panamanian sequences and world-wide HIV-1 sequences with the highest BLAST search similarity score were performed. Although BLAST search of Panamanian BC recombinants mainly retrieved CRF08_BC sequences from China (91%); Panamanian BC isolates were not phylogenetically related with that Asian CRF and branched with high support (posterior probability [*PP*]  =  0.99%) with one BC isolate from Dominican Republic (Caribbean) and one BC isolate from Spain (Europe) (Fig. 4A). BLAST search of the Panamanian BF recombinant retrieved BF sequences mainly isolated in Brazil (36%), Spain (24%) and the United States (16%) (Fig. 4B). Phylogenetic analysis showed that Panamanian BF sequence was positioned within a highly supported monophyletic cluster (*PP*  =  0.88) that was mainly (94%) composed by BF isolates from Brazil, including the CRF28_BF and CRF29_BF sequence references (Fig. 4B). BLAST search of Panamanian BG recombinant selected BG sequences mainly isolated in Cuba (82%) and Spain (16%) (Fig. 4C). The Panamanian BG sequence branched in a highly supported monophyletic cluster (*PP*  =  0.91) with CRF20_BG Cuban sequences (Fig. 4C). BLAST search of Panamanian subtype F1 sequence mainly recovered F1 and BF sequences from Brazil (72%) and Italy (18%). The phylogenetic analysis showed a close relationship between Panamanian and Brazilian subtype F1 samples (Fig. 4D). Finally, BLAST search of Panamanian subtype C isolate recovered sequences mainly isolated in India (38%), China (26%) and South Africa (24%) (Fig. 4E). Phylogenetic analysis showed that Panamanian subtype C sequence was more closely related to subtype C sequences from India than to sequences from other countries (Fig. 4E).

## Discussion

Nearly 29 years after the first diagnosed HIV case in Panama, this molecular epidemiology research represents the largest study to date on HIV subtype distribution in the country. An official report for HIV/AIDS in 2012 by the Ministry of Health (MINSA) showed a high percentage of AIDS cases (29.3%) and of asymptomatic carriers (48.7%) that do not define a transmission route [25], as we also observed in our data. This most likely reflects the difficulties of objectively capturing the sexual preference of both male and female subjects attending national health system clinics as subjects have the legal right of abstaining from giving this information [26]. As most of the epidemiological characteristics of the present study cohort are proportional to the officially reported HIV cases in the country; our study sample may adequately represent Panamanian HIV-1 molecular diversity.

In our study, we found that the Panamanian HIV epidemic is driven predominantly by HIV-1 subtype B, confirming the previous study [16]; but we also described for the first time the circulation of several non-B variants at very low prevalence (≤ 0.5%) that were classified as subtype F1, subtype C, CRF 20_BG, CRF28/29_BF and URFs_BC. Thus, the HIV-1 molecular epidemiology scenario in Panama is more complex than previously described [16], [17]; this is most likely due to the much larger number of sequences analyzed in our study.

The subtype F1 and the BF recombinant identified in this study were probably originated in Brazil. The identification of subtype F1 isolated from a heterosexual 47-year-old male was unexpected given the overall low prevalence of this subtype in the Americas, with exception of some Brazilian regions where it accounts for 10–20% of infections [27]–[33]. The BF recombinants are much more common in countries of the Southern Cone of South America [31]. A previous study described the detection of CRF12_BF, a recombinant characteristic of Argentina and Uruguay [34], in a Panamanian 38-year-old heterosexual male diagnosed in 1989 [16], [17]. The BF sequence identified in our study, by contrast, has a similar *pol* mosaic structure of CRF28/29_BF and was isolated from a 36-year-old male from the District of Panama diagnosed in 2009. This BF variant is particularly prevalent in the Brazilian city of Santos where it accounts for about 25% of infections [35], [36]. Of note, a recent study in Costa Rica also described the circulation of CRF17_BF and CRF29_BF [37], thus confirming a relative frequent influx of HIV-1 BF strains from South America into Central America.

The three Panamanian BC recombinants were isolated from one male and two females diagnosed in 1998, 2007 and 2009. Two of them were living in the province of Panama (cities of Panama and Arraiján) and one in the province of Colon (City of Colon). These two cities are the main ports for cruises and commercial trades and are located on opposite ends of the Panama Canal. Panamanian BC recombinant viruses detected in our study were phylogenetically related among each other and with two URFs_BC viruses detected in Spain [38] and Dominican Republic [39] in 2007 and 2005, respectively. The HIV-1 epidemic in Dominican Republic is dominated by subtype B and the URF_BC strain was described as a full-length genome [39]. Since sequences with a similar *pol* mosaic structure have been identified in at least six epidemiological unlinked subjects from three different countries, there is a possibility that there could be a new CRF_BC of Caribbean or Central American origin. Full-length genome sequencing of the HIV-1 BC Panamanian variants should be conducted to determine the URF or CRF status of those recombinant sequences.

The recombinant BG detected in Panama was a CRF20_BG-like virus isolated from a recently infected (in 2008) 25-year-old male who resided in the province of Chiriquí, near the Costa Rican border. Interestingly, recombinant BG has been also identified in a previous study from Costa Rica [37]. The CRF20_BG accounts for nearly 10–20% of HIV-1 infections in Cuba and is rarely observed outside this country [40]–[44], thus supporting a direct epidemiological link between CRF20_BG-like sequences from Panama and Cuba. Finally, the HIV-1 subtype C virus found in our study was phylogenetically related with the Indian subtype C lineage. A result that was agreed with our epidemiological data that the Panamanian subtype C virus was obtained from an Indian immigrant woman who was infected in her country of origin where subtype C is highly prevalent [45], [46].

The results from this study indicate an increasing introduction over the last 15 years of non-B subtype HIV-1 variants into Panama, particularly from South America (Brazil), the Caribbean (Cuba and Dominican Republic) and Asia (India). Panama has an 16.8% increase of foreign visitors in 2011 and 3.4% in stock of immigrants as percentage of population in 2010 [47], [48]. This increment is mainly explained by the improved governmental strategies that bring in tourists and by increases in construction of large-scale economic development projects that bring in immigrant workers [47], [49]. The greatest number of foreign travelers who visit Panama are most commonly from Colombia, China, the Dominican Republic, United States, Nicaragua, Costa Rica, Peru, Spain, Mexico and India [47]. Nevertheless, there have also been increases since 2008 in arrivals of visitors from South America, such as: Brazil (51.9%), Argentina (32.1%), Bolivia (41.5%), Ecuador (42.5%) and Venezuela (27.8%) [49], [50]. Furthermore, foreign residents with legal immigration status are mainly from South America (43.2%), Asia (15.7%), Central America (14.7%), North America (11.2%), the Caribbean (7.2%), and Europe (7.0%) [48]. Panama is a country of transit and commercial ports that trade and connect the Americas with Europe and Asia, which may potentiate the introduction of non-B variants. Of note, national epidemiological reports suggest that HIV epidemic is concentrated in high risk groups of MSM and sex workers [7], [25]. However, the impact of foreign travelers and immigrants in Panamanian HIV epidemic is unknown. Because of this, a cross-country epidemiological survey should be conducted in order to fully determine the HIV genetic diversity in most HIV infected populations, which would lead to improved HIV prevention programs.

Although our study only represents a small minority of the total estimated number of HIV subjects in the country, we demonstrate that a diverse non-B subtype variant circulates among subjects who required ARV drug resistance test. HIV viral diversity in the population being tested must be considered in selection of the viral load platform and ARV drug genotyping test. This nucleic acid or signal amplification assays rely on the use of sequence specific primers and/or probes. Therefore, HIV-1 increased heterogeneity may affect assay performance as the presence of natural polymorphisms in the target regions may reduce or inhibit hybridization thereby compromising the reliability of viral load quantification and genotyping test [51], [52]. Our “in-house” genotyping method was first designed using primers validated for subtype B. Successful amplification in most of the samples was only obtained with primers set accordingly to HIV-1 genetic diversity in our population. In fact, the five recombinants variants (BG, BC and BF) were successfully sequenced with our genotyping system. ViroSeq HIV-1 Genotyping System v2.0 has shown a decrease in performance on HIV-1 non-B strains in certain countries however, in Panama this diagnostic test has proven to perform well [52]. Recent studies evaluating viral load diagnostics test on current HIV genetic complexity found a lower correlations with subtypes C samples [53]. Therefore, the diverse non-B subtypes found in our study may play a significance influence on public health decision takers.

Understanding the continuous changes in the genetic profile of HIV epidemic is crucial since HIV genetic diversity has important implications for diagnostics, vaccine design, susceptibility to antiretroviral drugs, transmission capacity and virulence of the circulating virus, and disease progression [54]. The permanent survey of HIV genetic diversity in the infected Panamanian population over time is the first step in developing a full quantitative understanding of the processes that have shaped the Panamanian HIV epidemic and its evolution.

## Supporting Information

Figure S1 Schematic HIV-1 pol gene structure of the Panamanian recombinants BC samples (black circles) identified by NJ sub-region trees analyses according to breakpoints position defined. Bootstrap values greater than 75% are indicated.
(TIF)

Figure S2 Schematic HIV-1 pol gene structure of the Panamanian recombinants BF1 sample (black circle) identified by NJ sub-region trees analyses according to breakpoints position defined. Bootstrap values greater than 75% are indicated.
(TIF)

Figure S3 Schematic HIV-1 pol gene structure of the Panamanian recombinants BG sample (black circle) identified by NJ sub-region trees analyses according to breakpoints position defined. Bootstrap values greater than 75% are indicated.
(TIF)

Table S1 Subtypes reference strains used in the comparative analysis of the breakpoints positions, recombination analysis and phylogenetic analysis.
(DOCX)
