# Supplementary figures and images for: Molecular Epidemiology of HIV-1 in Panama: Origin of Non-B Subtypes in Samples Collected from 2007 to 2013
Source: PLoS One. 2014 Jan 13;9(1):e85153. doi: 10.1371/journal.pone.0085153 (PMC3890310; doi:10.1371/journal.pone.0085153)

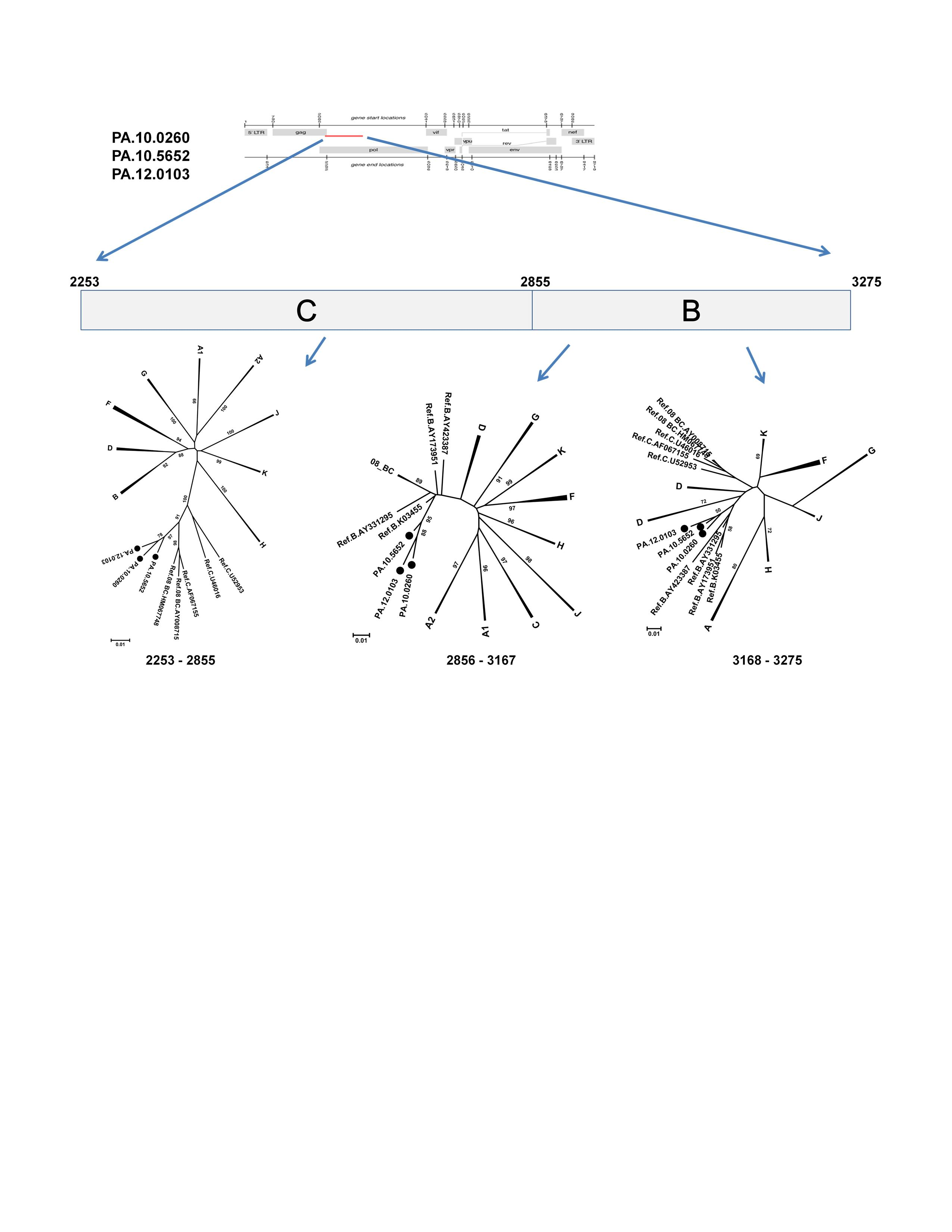

Supplement: Figure S1 — Schematic HIV-1 pol gene structure of the Panamanian recombinants BC samples (black circles) identified by NJ sub-region trees analyses according to breakpoints position defined. Bootstrap values greater than 75% are indicated. (TIF) [file pone.0085153.s001.tif]

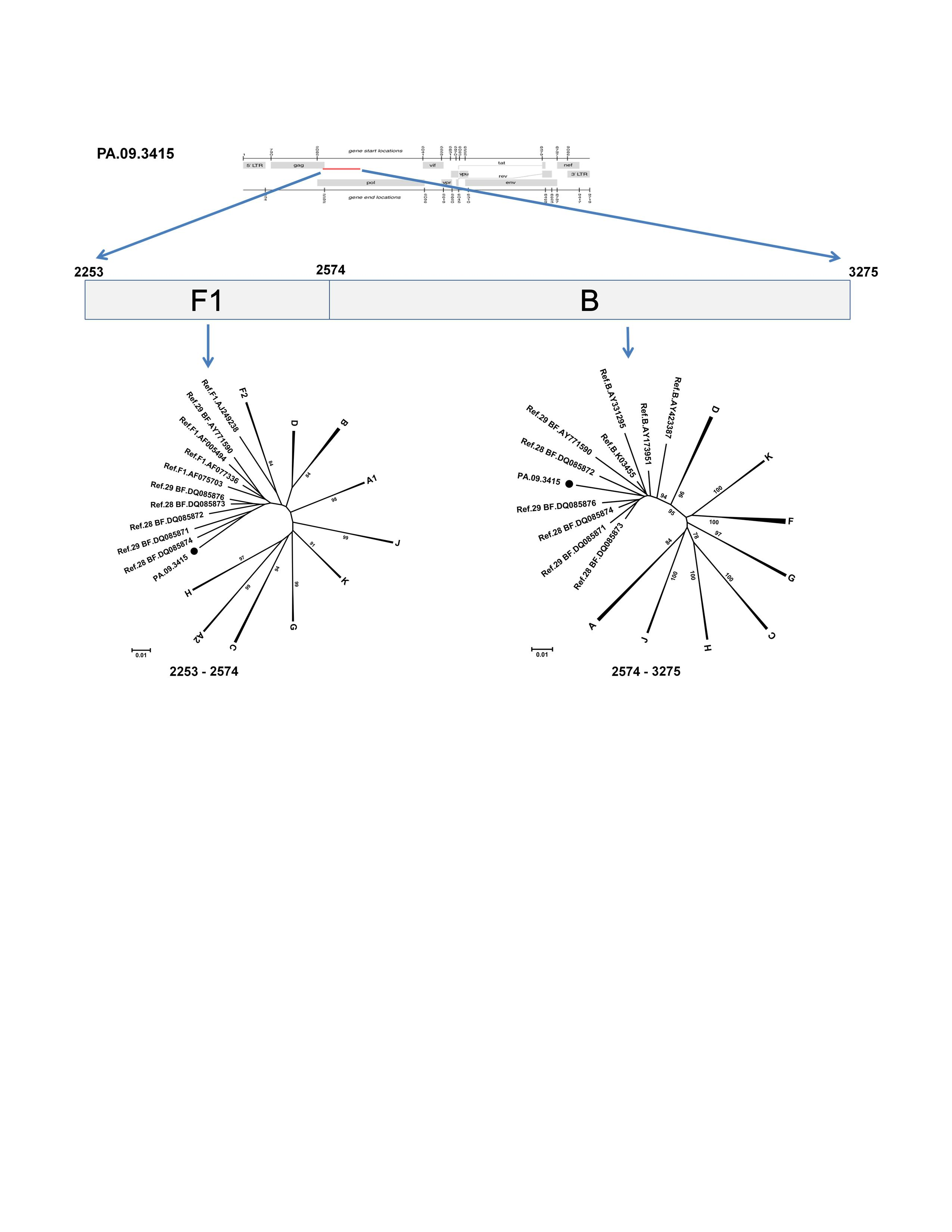

Supplement: Figure S2 — Schematic HIV-1 pol gene structure of the Panamanian recombinants BF1 sample (black circle) identified by NJ sub-region trees analyses according to breakpoints position defined. Bootstrap values greater than 75% are indicated. (TIF) [file pone.0085153.s002.tif]

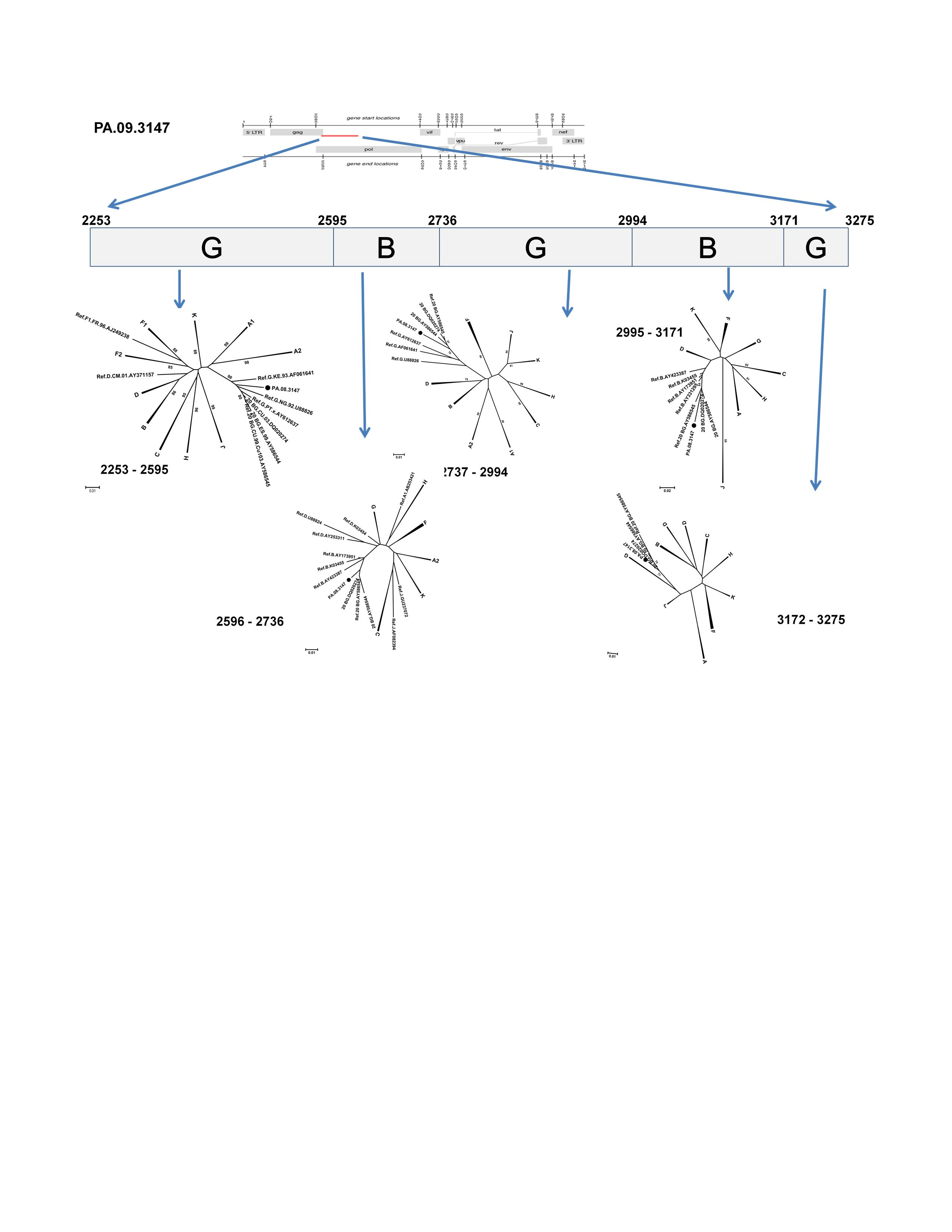

Supplement: Figure S3 — Schematic HIV-1 pol gene structure of the Panamanian recombinants BG sample (black circle) identified by NJ sub-region trees analyses according to breakpoints position defined. Bootstrap values greater than 75% are indicated. (TIF) [file pone.0085153.s003.tif]
