# Supplementary material for: Molecular Epidemiology of HIV-1 in Panama: Origin of Non-B Subtypes in Samples Collected from 2007 to 2013
Source: PLoS One. 2014 Jan 13;9(1):e85153. doi: 10.1371/journal.pone.0085153 (PMC3890310; doi:10.1371/journal.pone.0085153)
Supplement: Table S1 — Subtypes reference strains used in the comparative analysis of the breakpoints positions, recombination analysis and phylogenetic analysis. (DOCX) [file pone.0085153.s004.docx]

**Table S1**. Subtypes reference strains used in the comparative analysis of the breakpoints positions, recombination analysis and phylogenetic analysis.

| **Reference subtype name** | **Accession number** |
| --- | --- |
| Subtype A1 | AB253421 |
|  | AB253429 |
| Subtype A2 | AF286238 |
|  | GU201516 |
| Subtype B | AY423387 |
|  | K03455 |
|  | AY173951 |
|  | AY331295 |
| Subtype C | U52953 |
|  | U46016 |
|  | AF067155 |
| Subtype D | K03454 |
|  | AY371157 |
|  | AY253311 |
|  | U88824 |
| Subtype F1 | AF077336 |
|  | AF005494 |
|  | AF075703 |
|  | AJ249238 |
| Subtype F2 | AJ249236 |
|  | AJ249236 |
| Subtype G | U88826 |
|  | AF061641 |
|  | AY612637 |
| Subtype K | AJ249235 |
|  | AJ249239 |
| Subtype H | AF190127 |
|  | AF005496 |
| Subtype J | GU237072 |
|  | AF082394 |
